# Recurrent dizziness among adolescents in Denmark: Trends 1991–2022 and associations with sociodemographic factors, health, and health behaviours

**DOI:** 10.1007/s00431-025-06076-x

**Published:** 2025-03-13

**Authors:** Bjørn E. Holstein, Mogens Trab Damsgaard, Trine Pagh Pedersen, Mette Rasmussen, Mette Toftager, Katrine Rich Madsen

**Affiliations:** 1 University of Southern Denmark, National Institute of Public Health, Copenhagen, Denmark; 2 Department of Sports Science and Clinical Biomechanics, University of Southern Denmark, Odense, Denmark

**Keywords:** Adolescents, Dizziness, Epidemiology, HBSC, Trends

## Abstract

1) to study time trends in the prevalence of recurrent dizziness among adolescents in Denmark 1991–2022, and 2) to examine how dizziness was associated with sociodemographic factors, mental health related factors, health status, and health behaviours in 2022. The study focused on recurrent dizziness, i.e. episodes of dizziness several times a week during the last six months. Data stem from the Danish arm of the international Health Behaviour in School-aged Children (HBSC) study which included 11-, 13- and 15-year-olds from random samples of schools in nine comparable surveys from 1991 to 2022, *n* = 40,102. We applied multivariate logistic regression analyses in the 2022 dataset (*n* = 5,737) to examine how dizziness was associated with other factors. In 2022, the prevalence of recurrent dizziness was 14.3% (boys 8.8%, girls 19.7%), significantly higher than the 7.1% in the surveys 1991–2018. The prevalence in 2022 was significantly higher among girls, older students, and students not living with both parents. Dizziness was significantly elevated among students with short sleep duration, who skipped breakfast, used alcohol and tobacco, felt lonely, had low life satisfaction, low self-esteem, were exposed to bullying at school, felt high schoolwork pressure, low school satisfaction, who were underweight, overweight, had poor self-rated health, chronic illness, injuries in the last year, headache, stomachache, backpain, feeling low, irritability/bad temper, nervousness, difficulties falling asleep, and poor/restless sleep. *Conclusion*: The study suggested that dizziness is a general indicator of not feeling well, run down, or suffering rather than a sign of specific somatic health problems.

**Table:** 

**What is Known:** • *Recurrent dizziness is common among adolescents and may limit daily activities and harm quality of life.* • *Recurrent dizziness in adolescence may reflect somatic and mental health problems and is strongly associated with headache.*
**What is New:** • *The prevalence of recurrent dizziness among adolescents in Denmark was stable 1991–2018 and increased steeply from 2018 to 2022.* • *Recurrent dizziness was strongly associated with poor health behaviours, a broad range of somatic and mental health problems, and exposure to stressors.*

**What is Known:**

• *Recurrent dizziness is common among adolescents and may limit daily activities and harm quality of life.*

• *Recurrent dizziness in adolescence may reflect somatic and mental health problems and is strongly associated with headache.*

**What is New:**

• *The prevalence of recurrent dizziness among adolescents in Denmark was stable 1991–2018 and increased steeply from 2018 to 2022.*

• *Recurrent dizziness was strongly associated with poor health behaviours, a broad range of somatic and mental health problems, and exposure to stressors.*

## Introduction

Dizziness, often referred to as vertigo, is common among adolescents and encompasses sensations such as light-headedness, balance problems, and a spinning feeling [1–4]. Recurrent dizziness and vertigo often limit daily activities [2], can harm the adolescent’s quality of life. and cause emotional problems [5–8]. Most cases are benign, e.g. dizziness after getting up quickly, but chronic or recurrent dizziness may need medical attention.

Dizziness among adolescents may reflect somatic problems such as sequela after concussion, whiplash or other trauma. It may stem from epilepsy, inflammation of the middle ear, disorders of the inner ear, motor disorders of the visual system, vestibular migraine, psychogenic and systemic diseases, hypoglycemia and fear of height disorder [1, 8–14]. Dizziness is related to migraine [15, 16] and may be a sign of mental disorders [5, 17]. It is associated with perceived stress and exposure to stressors in daily life [15, 18].

There are few studies of the prevalence of dizziness among adolescents and the findings vary substantially depending on age group and the question asked, e.g. whether to report single episodes or recurrent dizziness [1, 2, 4, 17, 19]. Castillo-Bustamante et al. [5] reported that 8–10% of children and adolescents suffer from vertigo. There are no studies of trends over time, and few studies of associations with sociodemographic factors, health status and health behaviors. The available studies showed that dizziness was more common among girls than boys [15, 20] and increased with age [19]. Dizziness was associated with late pubertal status, headache, musculoskeletal pain [1–3, 7, 9, 11, 15, 16, 21–24], cognitive and psychiatric problems [3, 5, 17, 18]. Dizziness in adolescence was not associated with socioeconomic status [2].

The aim of this study was 1) to present time trends in the prevalence of recurrent dizziness among adolescents in Denmark 1991–2022, and 2) to examine how recurrent dizziness was associated with sociodemographic factors (e.g., age, gender, socioeconomic status), health behaviours (e.g., physical activity, sleep, smoking), mental health indicators (e.g., loneliness, life satisfaction, self-esteem) and health indicators and symptoms (e.g., weight status, aches, health complaints). We focused on recurrent dizziness defined as experiencing dizziness several times a week during the last six months.

## Materials and methods

### The trend sub-study

We used the Danish arm of the international Health Behaviour in School-aged Children (HBSC) study [25]. HBSC is a series of comparable cross-sectional surveys of representative samples of 11-, 13- and 15-year-olds conducted every fourth year in fifty countries. The Danish National HBSC Trend Datafile included a selection of variables from nine survey years, 1991, 1994, 1998, 2002, 2006, 2010, 2014, 2018 and 2022, spanning across 31 years. These nine surveys applied identical procedures for sampling, data collection and measurement. In each survey, we invited a random sample of schools, drawn from a complete list of public and private schools in Denmark. We invited all students in the fifth, seventh, and ninth grade, corresponding to the age groups 11, 13 and 15 years. The participating students answered the internationally standardized HBSC questionnaire at school [26]. The participation rate across all surveys was 84.9% of students enrolled in the participating classes, *n* = 41,143. We excluded 1,041 (2.5%) students with missing data on dizziness. The final study population was *n* = 40,102. Table 1 shows the participation rate, study population and distribution by sex and age group in the nine surveys.

**Table 1:** Participation rate, study population and distribution of sex and age group by survey year

	Survey year									
	1991	1994	1998	2002	2006	2010	2014	2018	2022	Total
Student participation rate ^a^	90.2	89.5	89.9	89.3	88.8	86.3	85.7	84.8	70.1	84.9
Students in the entire datafile	1860	4046	5205	4824	6269	4922	4534	3660	5823	41,143
Final sample size ^b^	1799	3920	5044	4750	6155	4835	4272	3590	5737	40,102
Pct. boys	49.7	49.4	49.5	48.6	49.1	50.0	48.4	49.6	49.8	49.3
Pct. 5th grade (11-year-olds)	31.1	31.5	33.9	36.5	37.7	37.4	32.5	40.6	36.0	35.1
Pct. 7th grade (13-year-olds)	34.9	34.9	35.8	33.6	35.5	33.6	34.8	33.7	35.9	34.9
Pct. 9th grade (15-year-olds)	34.0	33.6	30.3	29.9	26.8	29.0	32.7	25.2	28.7	29.9

^a^Participating student as percentage of total number of students in the participating classes

^b^Total number of students with data on sex, age group and dizziness

### The association sub-study

We used the most recent HBSC survey in 2022 [27], participation rate 70.1%, *n* = 5,823. After exclusion of 86 students (1.5%) with missing data on dizziness, the final study population was 5,737.

### Measurements

All data were self-reported. Dizziness was measured by one item from the HBSC Multiple Health Complaints Measure [26]: “In the past 6 months, how often have you experienced the following symptoms …? (eight items, including feeling dizzy). We dichotomized the responses into recurrent/several times a week ("about every day" and “more than once a week”) vs. less often ("about every week,” “about every month,” and “rarely or never”).

The study included five *sociodemographic factors*: 1) sex; 2) age group; 3) immigration background defined by country of birth for the student, his/her father and mother, categorized into Danish, descendants of immigrants, and immigrants; 4) family composition, categorized into living with two parents, living with one parent, and other family formats; and 5) socioeconomic status. Socioeconomic status was measured by father’s and mother’s labour market participation and occupation and coded in accordance with the Danish Occupational Social Class Measurement [28]. Each student was categorized by the highest-ranking parent into four groups: 1. High (e.g. professionals and managerial positions, large-scale business owners), 2. middle (e.g. technical and administrative staff, small-scale business owners, skilled workers), 3. low (unskilled and semi-skilled workers, economically inactive), and 4. unclassifiable.

The study included five *health behaviours* suggested as potential risk factors by Filippopulos et al. (2017) [15]: 1) Sleep duration on weekdays categorized into high (> 9 h per night), medium (6½−9 h) and low (6 h or less); 2) vigorous physical activity categorized into high (7 + hours/week), medium (1–6½ hours/week) and low (0-½ hour/week); 3) breakfast habits categorized into breakfast on all weekdays, 1–4 weekdays, and never; 4) alcohol use (only 15-year-olds) operationalized as number of times in one’s life having been really drunk, trichotomized into 0, 1–3, and 4 + times; 5) smoking (only 15-year-olds) categorized into non-smokers, occasional smokers, and daily smokers.

Inspired by Bigelow et al. (2020) [17], we included six *mental health indicators*: 1) Loneliness (never, sometimes, often/very often); 2) life satisfaction measured from 0 to 10 [29] and trichotomized into high (9–10), medium (6–8) and low (0–5); 3) self-esteem measured by the HBSC-DK self-esteem measure [30] (high, medium and low); 4) exposure to bullying at school (no exposure, occasional exposure, exposure at least twice a month); 5) perceived pressure from schoolwork (no pressure, a little/some pressure, a lot of pressure); 6) and school satisfaction (high, medium and low).

Inspired by other studies [3, 17, 23] we also examined how dizziness was associated with four *health indicators*: 1) Weight status using internationally standardized age- and sex-specific cutoff points for body mass index to define underweight grade 1–2, normal weight, and overweight + obesity [31, 32]; 2) self-rated health (very good, good, poor/fair); 3) self-reported chronic illness (no, yes); 4) and injuries treated by a health professional in the past 12 months (0, 1, 2 +). Moreover, we examined the association between dizziness and seven common symptoms measured by the HBSC Multiple Health Complaints Measure [26, 33]: headache, stomachache, backpain, feeling low, irritability or bad temper, feeling nervous, and difficulties falling asleep, trichotomized into seldom/never, up to once a week, more than once a week. Finally, we included frequency of poor or restless sleep (never, up to once a week, several times a week). The HBSC-study undergoes extensive pilot and validity studies [26]. Prior to the data collection in Denmark, we conducted pilot tests including focus group discussions with students to assess the face validity of the questionnaire.

### Statistical procedures

We used SAS version 9.4 for the analyses. *The trend sub-study* described prevalence of dizziness by survey year, separately for boys and girls and age-group. We used the Cochran-Armitage test to assess whether trends were significantly increasing/decreasing. This test assesses an association between a binary variable (here: ± recurrent dizziness) and an ordinal variable (here: survey year). *The association sub-study* used the 2022-survey and examined the associations between recurrent dizziness and other factors by logistic regression analyses, presenting odds ratio with 95% confidence interval (OR, 95% CI) for dizziness. The analyses were adjusted by sex and age group. We repeated the analyses with inclusion of interaction terms to examine whether sex and age group modified the associations between recurrent dizziness and other variables. Only statistically significant interactions were reported. The logistic regression analyses accounted for the applied cluster sampling by means of multilevel modelling (PROC GLIMMIX).

### Missing data

In most variables, the number of missing data was < 2%. Four variables had a high number of missing: weight status (*n* = 530, 9.2%), vigorous physical activity (*n* = 403, 7.0%), self-efficacy (*n* = 404, 7.0%), and poor/restless sleep (*n* = 360, 6.3%). In each analysis, we excluded participants with missing data.

## Results

### Prevalence and trends 1991–2022

**Fig. 1:**
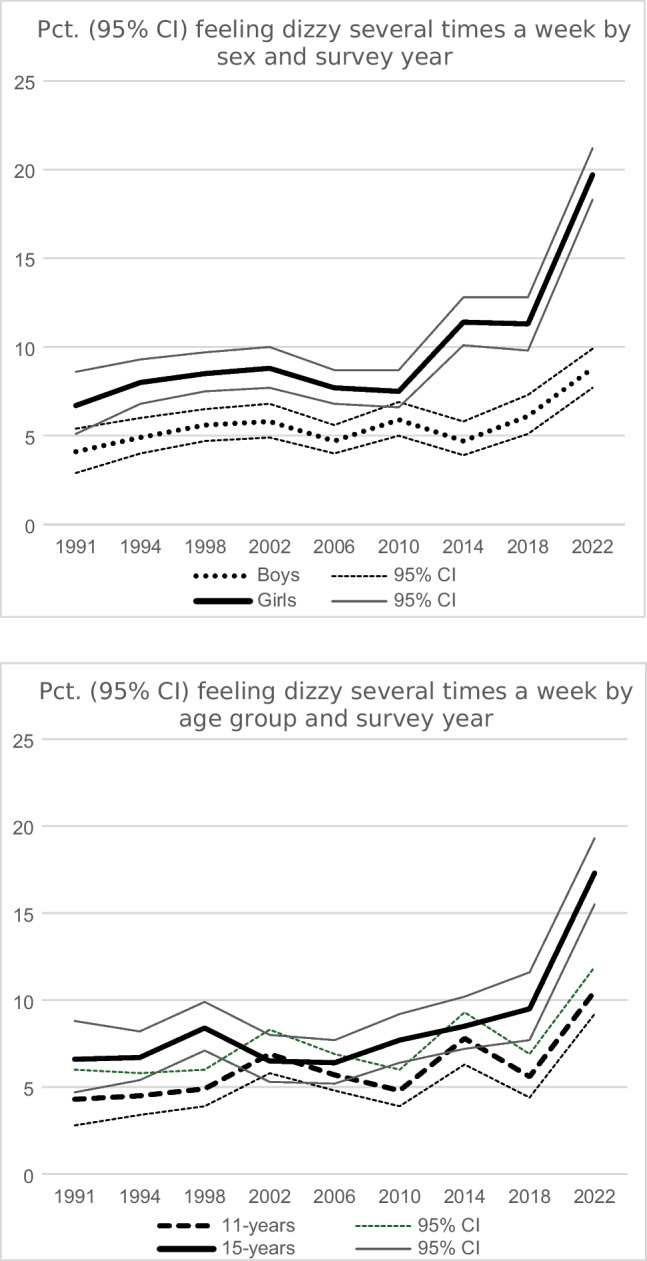
Pct. (95% CI) feeling dizzy several times a week 1991–2022 stratified by sex and age group

Table 2 shows the prevalence of recurrent dizziness by survey year, sex, and age group. In 2022, the prevalence was 14.3% (8.8% for boys and 19.7% for girls). Figure 1 shows the trends in recurrent dizziness from 1991 to 2022 stratified by sex and age group. The prevalence among girls was relatively stable from 1991 to 2010 whereafter it increased significantly to 2022 (Cochran-Armitage test, *p* < 0.0001). The prevalence for boys was relatively stable from 1991 to 2014, followed by a significant increase to 2022 (*p* < 0.0001). In most years, the confidence intervals for boys and girls were not overlapping, i.e. the sex-differences were statistically significant (Fig. 1). The confidence intervals for 11- and 15-year olds were not overlapping in 1991, 1994, 2018 and 2022, i.e. the prevalence was higher among the oldest students in these years (Fig. 1). There was a significant increase in the prevalence of recurrent dizziness in all age groups from 1991 to 2022 (*p* < 0.0001) (Table 2). The prevalence of *daily* dizziness (not shown in tab le) in 2022 was 5.3% (2.6% for boys and 8.0% for girls).

**Table 2:** Prevalence of recurrent dizziness by sex, age group and survey year

	Survey year									
	1991	1994	1998	2002	2006	2010	2014	2018	2022	*p*^a^
Total sample ^b^	5.5	6.5	7.1	7.4	6.2	6.7	8.2	8.7	14.3	< 0.0001
Boys	4.1	4.9	5.6	5.8	4.8	5.9	4.7	6.1	8.8	< 0.0001
Girls ^c^	6.7	8.0	8.5	8.8	7.7	7.6	11.4	11.3	19.7	< 0.0001
11-year-olds (5th grade) ^d^	4.3	4.5	4.9	6.9	5.7	4.9	7.7	5.6	10.5	< 0.0001
13-year-olds (7th grade)	5.4	8.0	8.0	8.5	6.8	8.0	8.2	11.9	15.6	< 0.0001
15-year-olds (9th grade)	6.6	6.7	8.4	6.5	6.4	7.7	8.6	9.5	17.3	< 0.0001

^a^p-value from Cochran-Armitage test

^b^The total prevalence of recurrent dizziness was 7.1% in the surveys from 1991 to 2018 and 14.3% in the survey in 2022, *p* < 0.0001

^c^The difference between boys and girls was statistically significant in all survey years, all p-values < 0.0001

^d^The difference between age groups was statistically significant in 2022, *p* < 0.0001

### Sociodemographic factors (Table 3)

In the total study population, the OR (95% CI) for dizziness increased by age group, 13-year-olds 1.60 (1.33–1.92), 15-year-olds 1.82 (1.49–2.19) compared to 11-year-olds. Tests for statistical interaction showed that the prevalence of recurrent dizziness increased by age among girls (*p* < 0.0001) but not boys (*p* = 0.2328). In addition to the association with sex and age group, the OR was significantly elevated among adolescents not living with two parents, 1.45 (1.11–1.89). Recurrent dizziness was not associated with immigration background or socioeconomic status.

**Table 3:** Adjusted^a^ OR (95% CI) for recurrent dizziness by sociodemographic factors and health indicators in 2022

Sociodemographic factors		OR (95% CI)	*p*
Sex^b^	Boys (*n* = 2855) Girls (*n* = 2882)	1 (ref.) **2.51 (2.18–3.00)**	< 0.0001
Age group^c^	11-year-olds (*n* = 2048) 13-year-olds (*n* = 2067) 15-year-olds (*n* = 1622)	1 (ref.) **1.60 (1.33–1.92)** **1.81 (1.49–2.19)**	< 0.0001
Immigration status	Danish (*n* = 5128) Descendants (*n* = 357) Immigrants (*n* = 225)	1 (ref.) 0.87 (0.63–1.19) 1.26 (0.86–1.80)	0.3025
Family composition	Living with two parents (*n* = 4057) Living with one parent (*n* = 1094) Other family format (*n* = 431)	1 (ref.) **1.56 (1.31–1.87)** **1.45 (1.11–1.89)**	< 0.0001
Socioeconomic status	High (*n* = 2449) Medium (*n* = 1785) Low (*n* = 478) Unclassifiable (*n* = 1025)	1 (ref.) 0.89 (0.75–1.06) 1.08 (0.82–1.41) 0.87 (0.71–1.08)	0.4394
**Health behaviours**		**OR (95% CI)**	
Sleep quantity^b,c^	> 9 h per night (*n* = 1630) 6½−9 h per night (*n* = 3465) Max. 6 h per night (*n* = 642)	1 (ref.) **1.56 (1.25–1.95)** **2.88 (2.20–3.77)**	< 0.0001
Vigorous Physical Activity^c^	7 h a week (*n* = 929) 1–6 h a week (*n* = 3620) 0-½ hour a week (*n* = 785)	1 (ref.) 0.91 (0.73–1.14) 1.21 (0.92–1.59)	0.0280
Breakfast on weekdays^c^	All weekdays (*n* = 3602) 1–4 weekdays (*n* = 1247) Never (*n* = 910)	1 (ref.) **2.04 (1.70–2.45)** **2.58 (2.13–3.13)**	< 0.0001
Drunkenness, 15-year-olds	No experience (*n* = 628) 1–3 times (*n* = 578) 4 + times (*n* = 398)	1 (ref.) 1.35 (0.97–1.89) **2.95 (2.12–4.18)**	< 0.0001
Smoking, 15-year-olds	Non-smoker (*n* = 1411) Occasional smoker (*n* = 163) Daily smoker (*n* = 38)	1 (ref.) **2.31 (1.38–3.37)** **2.79 (1.44–6.04)**	< 0.0001
**Mental health indicators**		**OR (95% CI)**	
Loneliness	Never (*n* = 3564) Sometimes (*n* = 1696) Often/very often (*n* = 477)	1 (ref.) **2.12 (1.78–2.52)** **5.55 (4.43–6.94)**	< 0.0001
Life satisfaction	High (*n* = 1617) Medium (*n* = 3294) Low (*n* = 804)	1 (ref.) **2.12 (1.66–2.70)** **8.12 (6.24–10.56)**	< 0.0001
Self-esteem	High self-esteem 3 (*n* = 3302) Medium self-esteem 1–2 (*n* = 1412) Low self-esteem 0 (*n* = 1023)	1 (ref.) **2.38 (1.97–2.88)** **3.99 (3.29–4.85)**	< 0.0001
Exposure to bullying at school	No exposure (*n* = 4668) Occasionally (*n* = 708) At least twice/month (*n* = 361)	1 (ref.) **2.13 (1.73–2.63)** **3.30 (2.57–4.24)**	< 0.0001
Perceived pressure from schoolwork	No pressure (*n* = 1285) A little/some (*n* = 3793) A lot (*n* = 600)	1 (ref.) **1.70 (1.34–2.16)** **6.19 (4.69–8.16)**	< 0.0001
School satisfaction	High (*n* = 1452) Medium (*n* = 3259) Low (*n* = 974)	1 (ref.) **1.98 (1.57–2.49)** **5.22 (4.05–6.73)**	< 0.0001
**Health indicators/symptom**		**OR (95% CI)**	
Weight status	Thinness grade 1–2 (*n* = 245) Normal weight (*n* = 4391) Overweight + obese (*n* = 622)	**1.62 (1.17–2.25)** 1 (ref.) **1.29 (1.02–1.63)**	0.0021
Self-rated health	Very good (*n* = 1831) Good (*n* = 2975) Poor/fair (*n* = 877)	1 (ref.) **1.73 (1.40–2.14)** **5.72 (4.53–7.22)**	< 0.0001
Self-reported chronic illness	No (*n* = 4524) Yes (*n* = 1213)	1 (ref.) **2.86 (2.44–3.36)**	< 0.0001
Injuries in the past 12 months	None (*n* = 3021) One (*n* = 1280) Two or more (*n* = 1419)	1 (ref.) **1.35 (1.10–1.64)** **2.33 (1.96–2.79)**	< 0.0001
Headache	Seldom or never (*n* = 2511) Occasionally (*n* = 2163) More than once a week (*n* = 1050)	1 (ref.) **3.37 (2.65–4.29)** **15.11 (11.86–19.25)**	< 0.0001
Stomachache^b^	Seldom or never (*n* = 2999) Occasionally (*n* = 2094) More than once a week (*n* = 609)	1 (ref.) **2.86 (2.34–3.49)** **12.39 (9.83–15.63)**	< 0.0001
Backpain	Seldom or never (*n* = 2815) Occasionally (*n* = 1896) More than once a week (*n* = 951)	1 (ref.) **2.15 (1.76–2.63)** **7.60 (6.20–9.32)**	< 0.0001
Feeling low	Seldom or never (*n* = 2320) Occasionally (*n* = 2328) More than once a week (*n* = 1089)	1 (ref.) **2.13 (1.70–2.67)** **8.64 (6.84–10.91)**	< 0.0001
Irritability / bad temper	Seldom or never (*n* = 1252) Occasionally (*n* = 2870) More than once a week (*n* = 1615)	1 (ref.) **2.50 (1.76–3.54)** **12.27 (8.73–17.26)**	< 0.0001
Feeling nervous	Seldom or never (*n* = 1802) Occasionally (*n* = 2696) More than once a week (*n* = 1239)	1 (ref.) **2.08 (1.62–2.69)** **9.22 (7.16–11.89)**	< 0.0001
Difficulties falling asleep	Seldom or never (*n* = 2000) Occasionally (*n* = 1939) More than once a week (*n* = 1798)	1 (ref.) **1.65 (1.29–2.12)** **6.72 (5.38–8.39)**	< 0.0001
Poor/restless sleep^b^	Never (*n* = 1298) Once/week or less often (*n* = 3182) Several times a week (*n* = 897)	1 (ref.) **1.92 (1.49–2.49)** **7.28 (5.54–9.57)**	< 0.0001

^a^Adjusted for sex and age group

^b^Statistically significant interaction with age group

^c^Statistically significant interaction with sex

Estimates in bold are statistically significant

### Health behaviours (Table 3)

In the total study population, there was no association between dizziness and vigorous physical activity. Test for statistical interaction showed that the odds ratio for recurrent dizziness increased by decreasing vigorous physical activity among girls (*p* < 0.0118) but not boys (*p* = 0.0648). The OR for recurrent dizziness decreased with sleep quantity; the OR (95% CI) for those with the shortest sleep was 2.88 (2.20–3.77), and the association was significantly steeper among girls than boys. The OR for recurrent dizziness for those who always skipped breakfast on weekdays was 2.58 (2.13–3.13). The association was significantly steeper for girls. There was a significantly elevated OR of recurrent dizziness among adolescents who had experiences with being drunk at least four times, 2.95 (2.12–4.18), and who were occasional (2.31 (1.38–3.37)) or daily smokers, 2.79 (1.44–6.04).

### Mental health indicators (Table 3)

There was a strong and graded association between recurrent dizziness and each of the six mental health indicators (loneliness, low life satisfaction, low self-esteem, exposure to bullying at school, perceived pressure from schoolwork, and low school satisfaction). Some of these associations were remarkably strong, for example students with low life satisfaction had eight times higher odds of experiencing recurrent dizziness than those with high life satisfaction (OR 8.12, 95% CI 6.24–10.56).

### Health indicators and symptoms (Table 3)

The OR (95% CI) for recurrent dizziness was significantly elevated among students with grade 1–2 thinness (1.62, 1.17–2.25) and students with overweight/obesity (1.29, 1.02–1.63). The OR was also significantly elevated for students with poor self-rated health, 5.72 (4.53–7.22), students with two or more injuries in the last 12 months, 2.33 (1.96–2.79), and students with self-reported chronic illness, 2.86 (2.44–3.36). Finally, there was a strong and graded association between recurrent dizziness and eight health complaints (headache, stomachache, backpain, feeling low, irritability/bad temper, feeling nervous, difficulties falling asleep, and poor/restless sleep. For example, the OR (95% CI) for recurrent dizziness was 2.86 (2.34–3.49) among participants with occasional stomachache and 12.39 (9.83–15.63) among participants with stomachache more than once a week. The association between dizziness and poor/restless sleep was statistically significant for both boys and girls but significantly steeper among girls. The association between recurrent dizziness and frequency of stomachache was significant in all age groups but significantly steepest in the youngest age group.

## Discussion

We aimed to present time trends in the prevalence of recurrent dizziness among adolescents from 1991 to 2022, and to examine how dizziness was associated with sociodemographic factors, health behaviours, indicators of mental health, and health indicators/symptoms in 2022. The study focused on recurrent dizziness defined as more than weekly during a six month period.

There were five main findings: *First,* the prevalence of recurrent dizziness was stable around 7% in the 1990s and the first decade of this century, whereafter it rose significantly from 2010 to 2022 among girls, from 2018 to 2022 among boys, from 2010 to 2022 among 13- and 15-year olds, and from 2018 among 11-year-olds. This trend has a remarkable similarity with the sudden steep increase in mental health problems such as feeling low, feeling nervous, difficulties falling asleep, irritability/ bad temper, loneliness, low life satisfaction, perceived school pressure, tiredness, and low social competence between 2010 and 2022 in Denmark [27]. Similar trends were found in many European countries [34, 35].

The prevalence of recurrent dizziness in 2022 (8.8% for boys and 19.7% for girls) was like a UK study which found that 13.1–20.6% of children reported experiencing dizziness between one and four times a week [2]. The prevalence in our study was higher than the 5.3% reported by Li et al. [19] and lower than the 72.0% reported by Langhagen et al., the latter study counting at least one episode of dizziness/vertigo in the last three months [4]. Few studies have examined time trends in adolescent dizziness. The relevant studies included dizziness in composite measures of psychosomatic complaints [34, 35]. These studies confirm a steep increase in psychosomatic complaints from approximately 2010 but do not report specific trends in dizziness.

*Second*, recurrent dizziness was significantly more common among girls than boys, as showed in other studies [15, 20]. A new finding was that not only did girls report more dizziness than boys, but associations between dizziness and poor health behaviours (low physical activity, short sleep, skipping breakfast) were significantly stronger in girls than boys. The prevalence of recurrent dizziness was higher among older adolescents, as also showed by Li et al. [19]. The prevalence was higher among adolescents not living with two parents. There was no association with immigration background and socioeconomic status, which corresponds with the study by Humphriss & Hall [2].

*Third*, recurrent dizziness was significantly associated with health behaviours such as short sleep duration, skipping breakfast, having been drunk and smoking. Filippopulos et al. also found that dizziness in adolescence was associated with harmful health behaviours [15]. *Fourth*, recurrent dizziness was strongly associated with mental health indicators (loneliness, low life satisfaction, low self-esteem, exposure to bullying at school, perceived pressure from schoolwork, and low school satisfaction). Other studies confirm the association between dizziness and mental health problems [3, 5, 6, 8, 15, 17, 18]. *Fifth,* recurrent dizziness was significantly associated with all included health indicators and symptoms. Other studies confirmed the strong association between dizziness and headache [1–3, 7, 11, 15, 16, 21, 22], especially migraine, but recurrent dizziness was just as strongly associated with other aches. Two other studies also reported an association between dizziness and musculoskeletal pain [24] and poor sleep quality [12].

In summary, recurrent dizziness is associated with so many indicators of stress and lack of well-being that it seems to be a general indicator of not feeling well. Recurrent dizziness may also reflect specific and serious somatic health problems. Both mental health problems and specific somatic problems should be taken seriously by health care professionals and result in a careful diagnostic effort, including several strands of insight and expertise. Clinical experiences confirm that adolescents with a history of migraine, nausea, intolerance to head movements, anxiety and depression, and external stressors are more predisposed to developing recurrent dizziness [4, 5]. It is important to acknowledge clusters with vertigo/dizziness symptoms and other health problems, and clusters of different vertigo syndromes [4]. According to Castillo-Bustamante et al. [5], an important challenge in the evaluation of dizziness in adolescents is the increasing rate of somatoform and psychiatric disorders.

### Strengths and limitations

The strengths of the study include the large and unselected study population, and the uniform trend data covering an extended time-period. One important limitation is the cross-sectional study-design, so it is not possible to determine the causality between dizziness and the included variables. Neither are we able to explain the substantial increase in recurrent dizziness in recent years, for instance whether changes in adolescent lifestyles, screen time, or school pressure contribute to this increase. The cross-sectional design of each survey prevents us from determine whether recurrent dizziness was a precursor of or a result of e.g. lifestyle factors.

Another limitation is that measurement of dizziness. Dizziness is an unclear term as it may reflect vertigo, balance problems, feeling faint, woozy, and light-headed. There are few validation studies of our dizziness measure. The HBSC Multiple Health Complaints Measure is reliable assessed by consistent response patterns and valid assessed by qualitative interviews [36, 37] and analysis of construct validity [33, 38]. However, there are better ways to measure dizziness, e.g. balance assessment [2] and measurements that categorize dizziness by etiology and clinical manifestations [4, 15, 39]. We recommend use of such elaborated measurements in future studies.

### Implications

From a research point of view, we recommend further studies to produce more exact and appropriate dizziness prevalence among children and adolescents. We also recommend longitudinal studies such as the study by Humphriss et al. to sort out whether dizziness is a risk factors for psychosocial and health outcomes or an outcome of psychosocial and health problems [2]. Devaraja [10] highlights the unclear way adolescents present their dizziness symptoms. Qualitative studies may show how adolescents experience, explain and report problems like vertigo, dizziness, and feeling woozy or light-headed. We also need to know whether the reporting of dizziness is embedded in broader cultural habits.

Several scholars suggest that clinicians need more insight into dizziness among adolescents and need a more systematic approach to diagnosis and treatment [1, 3, 9, 13]. The diagnostic process is difficult due to complex etiology and unclear manifestations and there is a need for collaboration between several medical disciplines to establish diagnosis and find appropriate treatment [3, 7, 10, 21, 39, 40].

## Conclusion

The prevalence of recurrent dizziness among 11–15-year-olds in 2022 was 8.8% for boys and 19.7% for girls. The prevalence increased significantly from 2010 to 2022. Recurrent dizziness was associated with harmful health behaviours, mental health problems, and indicators of poor health. The study suggested that dizziness may be a general indicator of not feeling well, run down, or suffering, rather than a sign of specific somatic health problems.

## Data Availability

Applications to access the dataset should be sent to the Primary Investigator of the Danish HBSC Study, Dr. Katrine Rich Madsen, krma@sdu.dk.

## Abbreviations

HBSC: Health Behaviour in School-aged Children
OR 95% CI: Odds Ratio (95% Confidence Interval)
SAS: Statistical Analysis System
